# Diagnostic consistency of GLIM criteria and PG-SGA for malnutrition in patients with pancreatic cancer

**DOI:** 10.3389/fnut.2025.1642171

**Published:** 2025-10-08

**Authors:** Feifei Wang, Cong Wang, Liwei Zhang, Xiuli Li

**Affiliations:** ^1^Department of Oncology, The First Hospital of Hebei Medical University, Shijiazhuang, China; ^2^Department of Nursing, The First Hospital of Hebei Medical University, Shijiazhuang, China

**Keywords:** GLIM criteria, PG-SGA, pancreatic cancer, malnutrition, diagnostic consistency, nutritional assessment

## Abstract

**Objective:**

This study aimed to evaluate the diagnostic consistency between the Global Leadership Initiative on Malnutrition (GLIM) criteria and the Patient-Generated Subjective Global Assessment (PG-SGA) for identifying malnutrition in patients with pancreatic malignant tumors.

**Methods:**

A total of 108 pancreatic cancer patients from our hospital with a Nutritional Risk Screening (NRS) 2002 score ≥ 3 were enrolled. Demographic and clinical data were collected. Nutritional risk was assessed using NRS 2002, while malnutrition was evaluated by PG-SGA and GLIM criteria. The diagnostic consistency between GLIM and PG-SGA was analyzed using Cohen’s kappa (*κ*) and prevalence-adjusted bias-adjusted kappa (PABAK), including a subgroup analysis of malnutrition severity. Pearson correlation examined the relationship between these tools and conventional nutritional indicators, while T-tests were used to compare functional outcomes (handgrip strength) and clinical outcomes (length of stay) between nutritional groups.

**Results:**

The mean NRS 2002 score was 3.37 ± 0.98, with 75.0% (81/108) of patients identified as being at nutritional risk. Significant differences in nutritional risk were observed based on age, Body Mass Index (BMI), tumor stage, and tumor size (*p* < 0.05). Malnutrition prevalence was 60.2% (65/108) according to GLIM criteria and 63.9% (69/108) according to PG-SGA. The consistency analysis between GLIM and PG-SGA yielded a Cohen’s kappa value of 0.71 (*p* < 0.01), indicating substantial agreement. The PABAK was 0.78, confirming substantial agreement after adjusting for prevalence effects. The agreement for malnutrition severity (GLIM Stage 1/2 vs. PG-SGA Moderate/Severe) was moderate (*κ* = 0.58). Both GLIM and PG-SGA scores demonstrated significant positive correlations with arm circumference (AC), calf circumference (CC), BMI, serum albumin (Alb), and hemoglobin (Hb) levels (*p* < 0.05). Furthermore, malnutrition diagnosed by either GLIM or PG-SGA was significantly associated with lower handgrip strength and longer hospital stays (*p* < 0.01).

**Conclusion:**

Patients with pancreatic malignant tumors exhibit a high prevalence of nutritional risk and malnutrition. The GLIM criteria and PG-SGA demonstrate good consistency in diagnosing malnutrition in this patient population. Both tools effectively identify patients with functional deficits and poorer clinical outcomes, supporting the utility of GLIM as a practical assessment tool in clinical settings.

## Introduction

Pancreatic cancer is a highly aggressive malignancy of the digestive system, characterized by its insidious onset, rapid progression, and profound systemic effects, often leading to a wasting disease state (1). A significant majority, approximately 80–90% of patients, experience symptoms such as emaciation, fatigue, and unintentional weight loss even in the early stages of the disease (2). As the tumor advances, patients frequently develop cachexia, electrolyte imbalances, and hypoproteinemia, underscoring the critical importance of nutritional support in the comprehensive management of pancreatic cancer (3). However, the hypermetabolic state induced by the tumor, coupled with impaired digestion and absorption due to organ involvement and potential biliary obstruction, makes timely and accurate nutritional screening and assessment a cornerstone of effective nutritional intervention (4, 5).

Currently, the Nutritional Risk Screening 2002 (NRS 2002) is widely utilized for initial nutritional risk screening in hospitalized patients, including those with malignancies (6). For individuals identified as being at nutritional risk, a more comprehensive nutritional assessment is warranted to grade malnutrition and guide therapeutic strategies. The Patient-Generated Subjective Global Assessment (PG-SGA) is an internationally recognized tool, endorsed by organizations such as the European Society for Clinical Nutrition and Metabolism (ESPEN) and the Academy of Nutrition and Dietetics, for detailed nutritional assessment in oncology patients (7). Despite its efficacy, the PG-SGA is a multi-component tool that can be time-consuming to administer (8).

In response to the need for a globally harmonized approach, the Global Leadership Initiative on Malnutrition (GLIM) criteria were developed to provide a standardized framework for diagnosing malnutrition in adults across various clinical settings (9). The GLIM criteria incorporate both phenotypic (e.g., weight loss, low BMI, reduced muscle mass) and etiologic (e.g., reduced food intake/assimilation, inflammation/disease burden) domains, offering a more streamlined assessment process (10). While GLIM’s convenience is an advantage, its specific performance and comparability to established tools like PG-SGA in the context of pancreatic cancer require further validation (11).

The consistency of malnutrition assessment—referring to the comparability and reliability of results obtained through different methods or by different evaluators—is a pivotal aspect of clinical nutrition research and practice (12). Given the multifactorial nature of malnutrition and the variety of assessment tools available, discrepancies between different instruments are possible, and a universal “gold standard” remains elusive. However, understanding the agreement between tools can help optimize diagnostic pathways. Therefore, this study aimed to evaluate the sensitivity, specificity, and overall diagnostic consistency of the GLIM criteria and PG-SGA in patients with pancreatic malignant tumors. The findings are intended to elucidate the clinical utility of GLIM in this specific high-risk population, thereby providing an evidence base for its integration into routine clinical practice.

## Materials and methods

### Study design and participants

This prospective observational study was conducted at our hospital, enrolling patients diagnosed with pancreatic cancer between January 2023 and December 2024. Inclusion criteria were: (1) histopathologically confirmed pancreatic malignant tumor; (2) age ≥ 18 years; (3) clear cognition and ability to communicate verbally; (4) NRS 2002 score ≥ 3, indicating nutritional risk requiring further assessment; (5) provision of written informed consent to participate. Exclusion criteria included: (1) presence of severe cardiac, hepatic, or renal comorbidities; (2) bedridden status precluding weight measurement; (3) contraindications to bioelectrical impedance analysis (e.g., implanted electronic devices, amputation); (4) concurrent diagnosis of other malignant tumors, particularly of the digestive system; (5) inability to cooperate with questionnaire completion or assessments.

### Data collection

Within 24 h of admission, the following data were collected by trained researchers using a standardized questionnaire and patient records:

Demographic and Clinical Data: This included gender, age, body mass index (BMI; calculated as weight (kg) / height (m^2^)), educational level, and occupation. Disease-related data comprised tumor stage (according to the American Joint Committee on Cancer, AJCC, staging system), tumor size (largest dimension in cm), and tumor location (e.g., head, body/tail of the pancreas).

### Nutritional screening and assessment

Nutritional Risk Screening 2002 (NRS 2002): As recommended by ESPEN, NRS 2002 was used for initial nutritional risk screening. It considers impaired nutritional status (weight loss, reduced food intake, low BMI), disease severity, and age. A total score ≥ 3 indicates nutritional risk (6).

Patient-Generated Subjective Global Assessment (PG-SGA): The PG-SGA was administered by trained clinical staff. It comprises patient-reported sections (weight history, food intake, symptoms, activities/function) and clinician-assessed sections (disease, metabolic demand, physical exam). Scores are categorized as: A (well-nourished, 0–1 points), B (moderately malnourished or suspected malnutrition, 2–8 points, with 2–3 often considered mild/moderate and 4–8 moderate), or C (severely malnourished, ≥9 points) (7, 13). For this study, a score ≥2 was considered indicative of malnutrition. For severity analysis, patients with scores of 2–8 were classified as ‘Moderate’, and those with scores ≥9 as ‘Severe’.

GLIM Criteria for Malnutrition Diagnosis: Malnutrition was diagnosed according to the GLIM consensus criteria (10, 14). This requires at least one phenotypic criterion and one etiologic criterion. *Phenotypic criteria:* (1) Non-volitional weight loss (>5% within past 6 months, or >10% beyond 6 months). (2) Low BMI (e.g., <18.5 kg/m^2^ if <70 years, <20 kg/m^2^ if ≥70 years; country-specific adjustments can be made). (3) Reduced muscle mass. This was assessed using a multi-frequency bioelectrical impedance analyzer (BIA) (InBody 770, InBody Co., Ltd., Seoul, Korea). Measurements were taken with the patient in a supine position after fasting for at least 8 h, emptying their bladder, and refraining from strenuous exercise for 12 h prior. Measurements were performed by two trained dietitians; the device was calibrated daily according to manufacturer instructions. The inter-observer coefficient of variation (CV) for ASMI was <5%, ensuring high reliability. Reduced muscle mass was defined by a low appendicular skeletal muscle mass index (ASMI), calculated as appendicular skeletal muscle mass (kg) / height (m)^2^, with cut-offs of <7.0 kg/m^2^ for men and <5.7 kg/m^2^ for women, in line with recommendations for Asian populations (15). *Etiologic criteria:* (1) Reduced food intake or assimilation (≤50% of energy requirements for >1 week, or any reduction for >2 weeks, or chronic GI conditions impacting absorption). (2) Inflammation/disease burden (acute disease/injury-related or chronic disease-related inflammation, often indicated by C-reactive protein or clinical signs of cancer). Severity is graded as moderate (stage 1) or severe (stage 2) based on the severity of the phenotypic criteria. Stage 1 (moderate malnutrition) is defined by a weight loss of 5–10% within 6 months, a low BMI of <18.5 (<70 years) or <20 (≥70 years), or mild-to-moderate muscle mass deficit. Stage 2 (severe malnutrition) is defined by a weight loss >10% within 6 months, a very low BMI (<17 or <18.5 respectively), or a severe muscle mass deficit.

### Anthropometric and laboratory measurements

Anthropometric measurements included height and weight (for BMI calculation), mid-upper arm circumference (AC), and calf circumference (CC), performed according to standardized protocols, with the average of three measurements recorded. Fasting venous blood samples (5 mL) were collected in the morning. Serum albumin (Alb) was measured using a biochemical analyzer, and whole blood hemoglobin (Hb) was determined using an automated cell analyzer.

### Functional and outcome measures

Handgrip strength (HGS) was measured as an indicator of muscle function using a calibrated Jamar hydraulic hand dynamometer (Sammons Preston, Bolingbrook, IL, USA). Patients were seated with their elbow flexed at 90 degrees, and three measurements were taken for the dominant hand, with the maximum value recorded. Low HGS was defined based on the European Working Group on Sarcopenia in Older People (EWGSOP2) criteria (<27 kg for men, <16 kg for women) (16). Length of hospital stay (LOS) was recorded from the patient’s electronic medical record as a clinical outcome measure.

### Statistical analysis

Data were entered into Excel 2019 and analyzed using SPSS version 26.0 (IBM Corp., Armonk, NY, USA). Continuous data are presented as mean ± standard deviation (SD) if normally distributed, or median (interquartile range) if not. Categorical data are presented as frequencies and percentages (n, %). Independent sample t-tests or Mann–Whitney U tests were used for comparing two groups of continuous data, as appropriate. Chi-square tests or Fisher’s exact tests were used for categorical data. Diagnostic consistency between GLIM and PG-SGA was assessed using Cohen’s kappa coefficient (*κ*) and prevalence-adjusted bias-adjusted kappa (PABAK). Kappa was interpreted as: <0.20 (poor), 0.21–0.40 (fair), 0.41–0.60 (moderate), 0.61–0.80 (good/substantial), and 0.81–1.00 (very good/almost perfect) (17). The potential influence of prevalence on the kappa statistic was noted as a consideration in interpretation (18). Sensitivity, specificity, and accuracy were calculated. Pearson correlation analysis was used to examine the relationship between nutritional assessment scores (or categorizations) and continuous nutritional parameters. A *p*-value < 0.05 was considered statistically significant.

## Results

### Patient characteristics and nutritional risk

A total of 108 patients with pancreatic malignant tumors were included. The mean NRS 2002 score for the cohort was 3.37 ± 0.98. Overall, 75.00% (81/108) of patients were identified as being at nutritional risk (NRS 2002 score ≥ 3). Significant differences in NRS 2002 scores and the proportion of patients at nutritional risk were observed across different age groups, BMI categories, tumor stages, and tumor sizes (all *p* < 0.05) (Table 1).

**Table 1:** Incidence of nutritional risk in enrolled patients (*n* = 108).

Characteristics	Number of cases	NRS 2002 score (points)	T/*F* value	*p* value (Score)	Nutritional risk cases [n(%)]	χ^2^ value	P value (Risk)
Gender			0.389	0.562		3.214	0.098
Male	57 (52.8%)	3.36 ± 0.19			41 (71.9%)		
Female	51 (47.2%)	3.43 ± 0.24			40 (78.4%)		
Age (years)			6.781	0.001		12.092	0.001
<40	19 (17.6%)	2.68 ± 0.23			8 (42.1%)		
40–60	35 (32.4%)	3.35 ± 0.11			23 (65.7%)		
≥60	54 (50.0%)	3.58 ± 0.20			50 (92.6%)		
BMI (kg/m^2^)			7.452	0.001		16.889	0.001
<18.5	8 (7.4%)	2.37 ± 0.15			5 (62.5%)		
18.5 ≤ BMI < 24.0	25 (23.1%)	2.46 ± 0.11			17 (68.0%)		
24.0 ≤ BMI < 28.0	46 (42.6%)	3.12 ± 0.36			34 (73.9%)		
≥28.0	29 (26.9%)	3.36 ± 0.72			25 (86.2%)		
Tumor staging			10.338	0.001		20.738	0.001
Stage I	12 (11.1%)	1.83 ± 0.19			7 (58.3%)		
Stage II	28 (25.9%)	2.99 ± 0.45			20 (71.4%)		
Stage III	41 (38.0%)	3.52 ± 0.33			33 (80.5%)		
Stage IV	27 (25.0%)	4.68 ± 0.45			21 (77.8%)		
Tumor size (cm)			14.685	0.001		23.412	0.001
≤3	38 (35.2%)	3.14 ± 0.29			24 (63.2%)		
>3	70 (64.8%)	4.39 ± 0.65			57 (81.4%)		

### Malnutrition diagnosis by GLIM criteria

According to the GLIM criteria, 65 out of 108 patients (60.19%) were diagnosed with malnutrition. The prevalence of malnutrition diagnosed by GLIM varied significantly with age, BMI category, tumor stage, and tumor size (*p* < 0.05) (Table 2).

**Table 2:** Malnutrition diagnosed by GLIM criteria in enrolled patients (*n* = 108).

Characteristics	Number of cases	Weight loss [n(%)]	Low BMI [n(%)]	Decreased muscle mass [n(%)]	Decreased food intake/assimilation [n(%)]	Disease burden/inflammation [n(%)]	Malnourished patients (GLIM) [n(%)]	χ^2^ value	*P* value
Gender								0.380	0.117
Male	57 (52.8%)	43 (75.4%)	32 (56.1%)	38 (66.7%)	46 (80.7%)	28 (49.1%)	35 (61.4%)		
Female	51 (47.2%)	39 (76.5%)	21 (41.2%)	38 (74.5%)	32 (62.7%)	23 (45.1%)	30 (58.8%)		
Age (years)								19.082	0.001
<40	19 (17.6%)	11 (57.9%)	9 (47.4%)	12 (63.2%)	10 (52.6%)	12 (63.2%)	6 (31.6%)		
40–60	35 (32.4%)	21 (60.0%)	16 (45.7%)	23 (65.7%)	26 (74.3%)	19 (54.3%)	21 (60.0%)		
≥60	54 (50.0%)	50 (92.6%)	28 (51.9%)	41 (75.9%)	42 (77.8%)	20 (37.0%)	38 (70.4%)		
BMI (kg/m^2^)								25.476	0.001
<18.5	8 (7.4%)	3 (37.5%)	2 (25.0%)	4 (50.0%)	6 (75.0%)	2 (25.0%)	2 (25.0%)		
18.5 ≤ BMI < 24.0	25 (23.1%)	14 (56.0%)	12 (48.0%)	18 (72.0%)	12 (48.0%)	7 (28.0%)	12 (48.0%)		
24.0 ≤ BMI < 28.0	46 (42.6%)	40 (87.0%)	28 (60.9%)	41 (89.1%)	41 (89.1%)	17 (37.0%)	32 (69.6%)		
≥28.0	29 (26.9%)	25 (86.2%)	11 (37.9%)	13 (44.8%)	19 (65.5%)	25 (86.2%)	27 (93.1%)		
Tumor staging								21.335	0.001
Stage I	12 (11.1%)	3 (25.0%)	4 (33.3%)	5 (41.7%)	8 (66.7%)	4 (33.3%)	5 (41.7%)		
Stage II	28 (25.9%)	18 (64.3%)	12 (42.9%)	20 (71.4%)	23 (82.1%)	16 (57.1%)	12 (42.9%)		
Stage III	41 (38.0%)	35 (85.4%)	21 (51.2%)	29 (70.7%)	29 (70.7%)	21 (51.2%)	17 (41.5%)		
Stage IV	27 (25.0%)	26 (96.3%)	16 (59.3%)	22 (81.5%)	18 (66.7%)	10 (37.0%)	10 (37.0%)		

### Malnutrition diagnosis by PG-SGA

Using the PG-SGA (score ≥2 indicative of malnutrition), 69 of 108 patients (63.89%) were identified as malnourished. Similar to GLIM, the prevalence of malnutrition diagnosed by PG-SGA was significantly associated with age, BMI category, tumor stage, and tumor size (*p* < 0.05) (Table 3).

**Table 3:** Malnutrition diagnosed by PG-SGA in enrolled patients (*n* = 108).

Characteristics	Number of cases	Malnourished patients (PG-SGA) [n(%)]	χ^2^ value	P value
Gender			0.956	0.446
Male	57 (52.8%)	37 (63.2%)		
Female	51 (47.2%)	32 (62.7%)		
Age (years)			18.790	0.001
<40	19 (17.6%)	5 (26.3%)		
40–60	35 (32.4%)	24 (68.6%)		
≥60	54 (50.0%)	41 (75.9%)		
BMI (kg/m^2^)			23.812	0.001
<18.5	8 (7.4%)	3 (37.5%)		
18.5 ≤ BMI < 24.0	25 (23.1%)	11 (44.0%)		
24.0 ≤ BMI < 28.0	46 (42.6%)	37 (80.4%)		
≥28.0	29 (26.9%)	23 (79.3%)		
Tumor staging			8.023	0.004
Stage I	12 (11.1%)	7 (58.3%)		
Stage II	28 (25.9%)	16 (57.1%)		
Stage III	41 (38.0%)	23 (56.1%)		
Stage IV	27 (25.0%)	12 (44.4%)		

### Consistency between GLIM criteria and PG-SGA

The overall consistency between GLIM criteria and PG-SGA in diagnosing malnutrition was substantial, with a Cohen’s kappa (*κ*) value of 0.71 (*p* < 0.01). To account for the high prevalence of malnutrition, the prevalence-adjusted bias-adjusted kappa (PABAK) was also calculated and found to be 0.78, confirming that the substantial agreement extends beyond the potential effects of prevalence. Good to moderate consistency was also observed when analyzing subgroups based on age, BMI, tumor stage, and tumor size (*κ* values ranging from 0.44 to 0.65, all *p* < 0.01) (Table 4).

**Table 4:** Diagnostic consistency between GLIM criteria and PG-SGA for malnutrition in patients with pancreatic cancer (*n* = 108).

Characteristics	Number of cases	Kappa	P value
Overall	108	0.71	<0.01
Gender			
Male	57 (52.8%)	0.57	<0.01
Female	51 (47.2%)	0.65	<0.01
Age (years)			
<40	19 (17.6%)	0.48	<0.01
40–60	35 (32.4%)	0.57	<0.01
≥60	54 (50.0%)	0.62	<0.01
BMI (kg/m^2^)			
<18.5	8 (7.4%)	0.48	<0.01
18.5 ≤ BMI < 24.0	25 (23.1%)	0.50	<0.01
24.0 ≤ BMI < 28.0	46 (42.6%)	0.56	<0.01
≥28.0	29 (26.9%)	0.62	<0.01
Tumor staging			
Stage I	12 (11.1%)	0.59	<0.01
Stage II	28 (25.9%)	0.62	<0.01
Stage III	41 (38.0%)	0.44	<0.01
Stage IV	27 (25.0%)	0.58	<0.01

### Consistency of malnutrition severity

A subgroup analysis was conducted to compare the consistency of malnutrition severity classifications between GLIM (Stage 1 vs. Stage 2) and PG-SGA (Moderate [score 2–8] vs. Severe [score ≥9]) among the 69 patients diagnosed as malnourished by PG-SGA. Among these patients, GLIM classified 45 (65.2%) as Stage 1 and 20 (29.0%) as Stage 2. PG-SGA classified 47 (68.1%) as moderately malnourished and 22 (31.9%) as severely malnourished. The cross-tabulation of severity diagnosis showed moderate agreement, with a Cohen’s kappa (*κ*) value of 0.58 (*p* < 0.01) (Table 5).

**Table 5:** Consistency of malnutrition severity between GLIM and PG-SGA (*n* = 69).

GLIM Severity	PG-SGA Severity		Total
	Moderate (Score 2–8)	Severe (Score ≥9)	
Stage 1 (Moderate)	35 (74.5%)	10 (45.4%)	45
Stage 2 (Severe)	12 (25.5%)	12 (54.6%)	24
Total	47	22	69

Kappa (κ) = 0.58; *p* < 0.01.

### Diagnostic accuracy of GLIM, PG-SGA, and NRS 2002

When considering PG-SGA as a clinical comparator, GLIM demonstrated a sensitivity of 86.8% and specificity of 99.3%. NRS 2002, as a screening tool, showed a sensitivity of 80.1% and specificity of 80.8% for identifying patients who were diagnosed as malnourished by the comprehensive assessment tools (PG-SGA/GLIM) used in this study. The Youden’s index was highest for GLIM (0.861). Area Under the Curve (AUC) values from ROC analysis indicated good diagnostic performance for all tools (Table 6).

**Table 6:** Diagnostic performance of GLIM, PG-SGA, and NRS 2002 for malnutrition assessment.

Methods	Sensitivity (%)	Specificity (%)	Positive predictive value (%)	Youden’s index	AUC (95%CI)	*P* value
GLIM	86.8	99.3	99.0	0.861	0.791–0.903	<0.01
PG-SGA	89.1	93.3	91.2	0.824	0.832–0.945	<0.01
NRS2002	80.1	80.8	82.5	0.609	0.826–0.936	<0.01

### Correlation with traditional nutritional indicators

Both GLIM-defined malnutrition status and PG-SGA scores exhibited significant positive correlations with anthropometric measures (AC, CC, BMI) and key laboratory parameters, including serum albumin (Alb) and hemoglobin (Hb) (All *p* < 0.05), suggesting that these assessment tools reflect objective clinical and physiological markers (Table 7).

**Table 7:** Correlation between GLIM/PG-SGA diagnosed malnutrition and traditional nutritional indicators.

Traditional nutritional indicators	GLIM		PG-SGA	
	R value	*P* value	*R* value	*P* value
BMI (kg/m^2^)	0.265	0.014	0.202	0.001
CC (cm)	0.238	0.009	0.221	0.012
AC (cm)	0.297	0.021	0.283	0.008
Alb (g/L)	0.301	0.008	0.211	0.009
Hb (g/L)	0.284	0.001	0.307	0.011

### Association with functional status and clinical outcomes

Malnutrition, as diagnosed by both GLIM and PG-SGA, was significantly associated with poorer functional status and clinical outcomes. Patients diagnosed with malnutrition by GLIM had significantly lower mean handgrip strength (21.3 ± 4.8 kg vs. 28.5 ± 5.2 kg; mean difference 7.2 kg, 95% CI 5.1–9.3; *p* < 0.01) and a longer mean hospital stay (18.9 ± 5.5 days vs. 12.4 ± 3.1 days; mean difference 6.5 days, 95% CI 4.2–8.8; *p* < 0.01) compared to well-nourished patients. Similarly, patients diagnosed as malnourished by PG-SGA showed significantly lower handgrip strength (22.0 ± 5.1 kg vs. 29.1 ± 5.0 kg; mean difference 7.1 kg, 95% CI 5.0–9.2; *p* < 0.01) and longer hospital stays (18.1 ± 5.2 days vs. 11.8 ± 2.9 days; mean difference 6.3 days, 95% CI 4.1–8.5; *p* < 0.01) (Table 8).

**Table 8:** Association between malnutrition status (GLIM/PG-SGA) and functional/clinical outcomes (*n* = 108).

Parameter	Category	Number of cases (n)	Handgrip strength (kg, mean ± SD)	*P* value	Length of stay (days, mean ± SD)	*P* value
GLIM diagnosis	Well-nourished	43	28.5 ± 5.2	<0.01	12.4 ± 3.1	<0.01
	Malnourished	65	21.3 ± 4.8		18.9 ± 5.5	
PG-SGA diagnosis	Well-nourished (score <2)	39	29.1 ± 5.0	<0.01	11.8 ± 2.9	<0.01
	Malnourished (score ≥2)	69	22.0 ± 5.1		18.1 ± 5.2	

Data were analyzed using the independent samples t-test; values are expressed as mean ± standard deviation (SD).

## Discussion

Pancreatic cancer is intrinsically linked with a high risk of malnutrition, which adversely impacts clinical outcomes, treatment tolerance, and quality of life (1, 19). Effective nutritional management, predicated on accurate and timely diagnosis of malnutrition, is therefore paramount. This study investigated the diagnostic consistency between the newer GLIM criteria and the well-established PG-SGA in patients with pancreatic malignant tumors, a population particularly vulnerable to nutritional decline.

Our findings reveal a substantial prevalence of nutritional risk (75.00% by NRS 2002) and malnutrition (60.19% by GLIM, 63.89% by PG-SGA) in this cohort, aligning with previous reports emphasizing the severe nutritional burden in pancreatic cancer patients (3, 20, 21). The PG-SGA, identified as an accurate and sensitive tool in numerous oncological settings (22, 23), demonstrated good diagnostic performance in our study, with results correlating significantly with objective nutritional markers like AC, CC, BMI, Alb, and Hb. This echoes findings by Ferreira et al. (20), who highlighted PG-SGA’s utility in gastrointestinal cancer patients. Some studies suggest that the PG-SGA’s detailed assessment of nutrition-impact symptoms allows it to effectively diagnose malnourished individuals, potentially identifying those with poorer prognoses who might be overlooked by initial risk screening alone. Specifically, the PG-SGA Short Form (PG-SGA SF) demonstrates high sensitivity (89%) and specificity (80%) as a malnutrition screening tool, with a cut-off score ≥5 showing strong agreement with the full PG-SGA assessment and significant association with 1-year mortality risk in ambulatory cancer patients, thereby highlighting its prognostic utility beyond basic screening (5, 24).

The GLIM criteria, designed for global application, identified a malnutrition rate of 60.19% in our pancreatic cancer cohort. This prevalence is within the range of 30–80% reported in various cancer populations using GLIM, with figures often higher in advanced or gastrointestinal malignancies (10, 25). For instance, some studies in advanced cancer reported GLIM-defined malnutrition rates between 62.2 and 80.0% (25). Our rate is slightly lower than the NRS 2002 risk identification, which is expected as NRS 2002 is a screening tool for risk, while GLIM diagnoses malnutrition. Omiya et al. (26) and Igarashi et al. (27) found varying GLIM-defined malnutrition rates (12.6–36.8%) in hepatobiliary-pancreatic or biliary tract cancers, potentially reflecting differences in patient populations (e.g., specific cancer types within HBP, stage, treatment status) and regional variations in GLIM operationalization, particularly muscle mass assessment (28). The high prevalence of advanced-stage disease in our cohort likely contributes to the observed malnutrition rates, as advanced cancer often correlates with increased metabolic derangements and cachexia (11).

A key finding of our study is the substantial agreement (*κ* = 0.71; PABAK = 0.78) between GLIM criteria and PG-SGA in diagnosing malnutrition in patients with pancreatic cancer. While the PG-SGA is a well-validated tool, we acknowledge it is not an absolute gold standard for malnutrition diagnosis. Its administration can be subjective and more time-consuming compared to other methods. While the PG-SGA is a comprehensive tool that relies on patient recall and clinician interpretation (averaging approximately 20 min per assessment in our experience), the GLIM assessment, which integrates objective BIA data, required less than 5 min to complete, highlighting its superior feasibility in busy clinical settings. The observed agreement, therefore, supports the construct validity of the more streamlined and objective GLIM framework in a population where PG-SGA is considered a robust benchmark (8, 12, 29). Furthermore, our analysis of malnutrition severity revealed moderate agreement (*κ* = 0.58), suggesting that while the tools are consistent in identifying malnutrition, their grading of its severity may diverge more frequently. This finding is consistent with recent literature indicating that GLIM and PG-SGA may weigh phenotypic and etiologic factors differently when assigning severity, warranting further investigation into the prognostic value of each tool’s severity staging, as evidenced by studies showing similar κ values and highlighting discrepancies in severity classification due to variations in muscle mass assessment and etiologic criteria weighting (30).

Crucially, our study extends beyond mere agreement by linking these diagnostic tools to objective functional and clinical outcomes. We found that malnutrition, as defined by either GLIM or PG-SGA, was significantly associated with lower handgrip strength and a longer hospital stay. This reinforces the clinical relevance of both tools, demonstrating that they effectively identify patients with functional impairments and who are likely to have poorer short-term outcomes. This observation is crucial, as it links these structured assessment tools to measurable physiological changes and healthcare utilization. The positive correlations reported in Table 7, showing that malnutrition status aligns with poorer anthropometric/biochemical values, further supports that these tools capture meaningful nutritional derangements. The correlation with serum albumin, a well-known marker of inflammation, is particularly relevant as it supports the ‘inflammation/disease burden’ etiologic criterion of GLIM, reinforcing the framework’s construct validity (14, 31). Some studies have noted that while PG-SGA is sensitive, GLIM may offer better specificity when a strict diagnostic confirmation is needed (29). This positions GLIM as a valuable tool for confirming malnutrition identified by initial screening or more subjective assessments.

This study has several strengths, including its prospective design and focus on a homogenous, high-risk cancer population. However, limitations should be acknowledged. Being a single-center study with a relatively modest sample size (*n* = 108), the results may not be generalizable to all settings. The sample size also limited the statistical power of our malnutrition severity subgroup analysis. Furthermore, our inclusion criteria, which restricted enrollment to patients with an NRS 2002 score ≥ 3, created a cohort already identified as being at nutritional risk. This pre-selection may inflate the observed prevalence of malnutrition and could potentially lead to an overestimation of the agreement between the GLIM criteria and PG-SGA. This factor should be considered when interpreting the kappa statistic and the overall consistency results. Lastly, while PG-SGA served as a robust comparator, no single “gold standard” for malnutrition exists, making direct validation challenging. The inclusion of functional outcome data (HGS) in our study helps to mitigate this by providing an objective anchor for the clinical validity of both tools. Future research should aim to validate these findings in a multicenter study and include patients with an NRS 2002 score < 3 to assess the general applicability of GLIM across the full spectrum of nutritional risk.

In conclusion, our study demonstrates a high prevalence of nutritional risk and malnutrition among patients with pancreatic malignant tumors. The GLIM criteria exhibit substantial diagnostic consistency with PG-SGA in this population, supporting GLIM’s applicability as a standardized, clinically relevant tool for malnutrition diagnosis. Its structured approach, combining phenotypic and etiologic criteria, along with its correlation with objective nutritional, functional, and clinical outcome markers, makes it a valuable instrument for routine nutritional assessment, facilitating timely and targeted nutritional interventions in these vulnerable patients.

## Data Availability

The original contributions presented in the study are included in the article/supplementary material, further inquiries can be directed to the corresponding authors.
